# Work Addiction Test Questionnaire to Assess Workaholism: Validation of French Version

**DOI:** 10.2196/mental.8215

**Published:** 2018-02-13

**Authors:** Hortense Ravoux, Bruno Pereira, Georges Brousse, Samuel Dewavrin, Thomas Cornet, Martial Mermillod, Laurie Mondillon, Guillaume Vallet, Farès Moustafa, Frédéric Dutheil

**Affiliations:** ^1^ Service Santé Travail Environnement Centre Hospitalier Universitaire de Clermont-Ferrand Clermont-Ferrand France; ^2^ Équipe Stress physiologique et psychosocial Laboratoire de Psychologie Sociale et Cognitive Université Clermont Auvergne, Centre National de la Recherche Scientifique Clermont-Ferrand France; ^3^ Service de Biostatistique Direction de la Recherche Clinique et de l'Innovation Centre Hospitalier Universitaire de Clermont-Ferrand Clermont-Ferrand France; ^4^ Service Addictologie Centre Hospitalier Universitaire de Clermont-Ferrand Clermont-Ferrand France; ^5^ Neuro-Psycho-pharmacologie des systèmes dopaminergiques sous-corticaux Université Clermont Auvergne Clermont-Ferrand France; ^6^ WittyFit Paris France; ^7^ Laboratoire de Psychologie et NeuroCognition Centre National de la Recherche Scientifique Université Grenoble Alpes Grenoble France; ^8^ Institut Universitaire de France Paris France; ^9^ Service des Urgences Centre Hospitalier Universitaire de Clermont-Ferrand Clermont-Ferrand France; ^10^ Faculty of Health Australian Catholic University Melbourne, Victoria Australia

**Keywords:** behavior, addictive, work, validation studies as topic, questionnaires, social welfare, health, public health

## Abstract

**Background:**

Work addiction is a significant public health problem with a growing prevalence. The Work Addiction Risk Test (WART) is the gold standard questionnaire to detect workaholism.

**Objective:**

The main objective of this study was to validate the French version of the WART.

**Methods:**

Questionnaires were proposed to voluntary French workers using the WittyFit software. There were no exclusion criteria. The questionnaire was administered anonymously for initial validity testing and readministered one week later for test-retest reliability. We also assessed the workers’ sociodemographic characteristics, as well as other measurements for external validity, such as stress, well-being, and coaddictions to tobacco, alcohol, and cannabis. Several psychometric properties of the French-WART were explored: acceptability, reliability (internal consistency [Cronbach alpha coefficient] and reproducibility [Lin concordance coefficient]), construct validity (correlation coefficients and principal component analysis), and external validity (correlation coefficients).

**Results:**

Among the 1580 workers using WittyFit, 187 (11.83%) agreed to complete the WART questionnaire. Of those, 128 completed the test-retest survey (68.4%). Acceptability found that all respondents had fully completed the questionnaire, with few floor or ceiling effects. Reliability was very good with a Cronbach alpha coefficient at .90 (internal consistency) and Lin concordance coefficient at .90 (95% CI .87-.94] with a difference on the retest of .04 (SD 4.9) (95% CI −9.6 to 9.7) (reproducibility). We identified three main dimensions (construct validity). Relationships between WART and stress and well-being confirmed its external validity.

**Conclusions:**

The French version of the WART is a valid and reliable instrument to assess work addiction with satisfactory psychometric properties. Used in occupational medicine, this tool would allow the diagnosis of work addiction and can be easily implemented in current practice.

## Introduction

Addiction to work, or workaholism, is defined as “a compulsion or an uncontrollable need to work incessantly” [1-3]. This pathology is in line with the general criteria of addiction, that is, preoccupation with an addictive object or behavior, mood modification, interpersonal conflict, withdrawal syndrome, tolerance, relapses, or continuation of this behavior despite the knowledge of its negative effects [2-5]. There is a real typology based on the major characteristics of individuals (the compulsive-dependent, the perfectionists, the achievement-oriented, the bulimic, the relentless, etc) [2,6,7]. It is important to differentiate workers suffering from workaholism from those who are engaged at work [4,8,9]. Workaholics are propelled by an obsessive inner drive they cannot resist, whereas engaged workers are intrinsically motivated [8,10]. Addiction to work results from the individual’s predisposition, sociocultural experiences, and behavioral reinforcements [1,2,9,11]. Addiction to work is a growing public health concern [1,11,12] with a prevalence ranging from 7.6% [13] to 22.2% [14] in European countries. Workaholics dedicate more time and energy to work than seems necessary [2,7,8]. This behavioral addiction would negatively affect the individual’s health and could lead to relationship problems (family conflicts, marital problems, impact on their children, and poor social relationship) [10,11,15], neuropsychic troubles (depression, burnout, sleep disorders, and general dissatisfaction) [2,4,8-10,15-17], consequences to professional life in the long term (lower productivity levels, absences, and strain at work) [1-3,11,17], and poorer physical health [11].

As we are being confronted with this growing health problem, it appears absolutely essential to possess validated tools. The Work Addiction Risk Test (WART) is a reference questionnaire for work addiction [2]. This test was developed by Robinson et al in 1999 [2,18-20] based on the experiences of clinicians treating workaholics [2]. We chose this tool because of its wide use (approximately 150 studies) and usability [2]. The English version of the WART has satisfactory psychometric properties [6,20]. *Reliability* is represented by *internal consistency* with a Cronbach alpha coefficient ranging from .85 [21] to .88 [18], and *reproducibility* with a test-retest correlation coefficient of .83 [20,22], and a Spearman-Brown split-half reliability coefficient of 0.85 [20]. *Construct validity* is built around five dimensions: compulsive tendencies, control, impaired communication and self-absorption, inability to delegate, and self-worth [20]. For *external validity*, work addiction was linked to a high level of stress [3,14,15,18,23] and a poor level of well-being [1-4]. In addition to work addiction, the same worker may also suffer from several addictions, such as consuming tobacco, cannabis, or alcohol [1,24]. To our knowledge, no studies have reported acceptability of the English version of WART.

The main objective of this investigation was the validation of the French version of the WART to allow its use in current practice. We aimed to evaluate its acceptability, reliability, construct, validity, and external validity. Stress, well-being, and coaddictions to tobacco, alcohol, and cannabis were used for evaluating external validity.

## Methods

### Recruitment

Questionnaires were proposed to voluntary French workers using the WittyFit software [25]. WittyFit is a Web platform that aims to improve the well-being at work, with a public-private partnership with the University Hospital of Clermont-Ferrand. Workers using WittyFit answer-validated questionnaires on behavioral data for baseline health profiling. The concept of WittyFit is to provide individualized feedback based on evidence-based medicine, with an aim to support behavioral change using a formal evaluation of changes in knowledge, practices, and health outcomes over time. The database is implemented from a human resource–generated number, which is then automatically converted into another number in the WittyFit database. Data provided by employers (such as from the professional roles or the occupational sector) are automatically associated with the human resource–generated number. All data are anonymous, and the name of the employee is never entered into the database. The study was approved by the National Commission for Data Protection and Liberties and by the South-East VI Ethics Committee (ClinicalTrials.gov NCT number NCT02596737). There were no exclusion criteria. The WittyFit users were informed of a forthcoming questionnaire validation study through this platform explaining the purpose of the study and the need to complete the questionnaire twice (test and retest, 1 week later).

### The Work Addiction Risk Test Questionnaire

The WART is a self-administered test with 25 statements for which the answers are scored *1-never true* to *4-always true* [20,26]. Respondents read the statements and mark their answers to describe their work habits [19,27]. The total score is the sum of the responses to the items—25 to 100—and the higher the score, the more one is considered addicted to work [18,21]. Scores from 25 to 56 were defined as “at low-risk of work addiction”; 57 to 66 as “at medium-risk of work addiction”, and from 67 to 100 as “at high-risk of work addiction” [12,18]. The WART consists of five dimensions, including compulsive tendencies (9 items: 3, 5, 6, 7, 8, 15, 18, 19, 20); control (7 items: 2, 4, 11, 12, 16, 17, 22); impaired communication and self-absorption (5 items: 13, 21, 23, 24, 25); inability to delegate (1 item: 1); and self-worth (2 items: 9 and 10) [1,15,20]. The first two dimensions are the key elements to differentiate workaholics [1].

### Translation of the Work Addiction Risk Test

In accordance with the literature [28], the following steps were performed for the validation of the French version of the WART: (1) translations of the WART into French performed by 2 independent native French translators; (2) back translation of the French version of the WART into English by 2 native English speakers, who had no knowledge of the original English version; (3) synthesis and comparison of all translations by a committee of experts, multidisciplinary and bilingual, to develop the final version of the WART; (4) validation study of the French version. The questionnaire was administered for the initial validity testing and readministered 1 week later for test-retest reliability. Workers received an individual alert through the WittyFit software to complete the surveys. The French version of the WART is presented in Multimedia Appendix 1.

### External Validity: Other Measurements

Well-being and perceived stress at work and at home were evaluated using visual analog scales (VAS) by moving a cursor on a horizontal, noncalibrated line, ranging from very low (0) on the left to very high (100) on the right [29-31]. Furthermore, tobacco, alcohol, and cannabis consumption were evaluated using 3 specific VAS—the number of cigarettes smoked per day from 0 to 30, the number of glasses of alcohol consumed per day from 0 to 8, and the number of cannabis consumption per month from 0 to 30.

### Statistical Analysis

The number of subjects required were determined in advance by following recommendations [32] and in accordance with our recruitment abilities. In this context, a complement of at least 120 participants appeared to be relevant for the test and 60 for the retest.

Statistical analysis was carried out using the Stata software version 13 (StataCorp). Qualitative data were described in size and associated frequencies, and the data were compared between groups—those who only completed the questionnaire once (*test*) and those who completed the survey twice (*test and retest*) —with the chi-square test or with the Fisher exact test. Quantitative data, expressed by the mean (SD) or the median (interquartile ranges) regarding the statistical distribution (the Shapiro-Wilk test), were compared between groups with the Student *t* test (or an analysis of variance [ANOVA]) or Mann-Whitney *U* test or the Kruskal-Wallis test, if the *t* test’s conditions were not respected (normality and homoscedasticity considered by the Bartlett test) for the quantitative variables. When appropriate (*P*<.05), a post hoc test for multiple comparison was deemed, namely, the Tukey-Kramer postANOVA test and Dunn test after the Kruskal-Wallis test. The comparisons between the groups for category parameters were achieved with the chi-square test or with the Fisher exact test. The difference was defined as statistically significant when the level of significance (*P*) was less than .05 (alpha risk=5%).

The psychometric properties of the WART were explored. We assessed the acceptability based on the calculation of missing data for each item and the dimension of the WART—data quality was deemed acceptable if less than 5% of data were missing. The accepted maximum for floor and ceiling effects was 15% [32]. The reliability of the French version was evaluated according to two criteria: (1) internal consistency based on the calculation of the Cronbach alpha coefficient (desirable values higher than .70-0.80) [32-39] and (2) reproducibility. The correlation coefficient (Pearson or Spearman, as per the statistic distribution) and Lin concordance coefficient were computed to assess test-retest reliability [40]. Values above .7 were considered satisfactory. An analysis using a mixed model (random effect subject time) and Bland and Altman’s graphic illustrations completed the analysis. The construct validity of the French version of the WART was explored by reviewing interitem and interdimensional correlations and using multidimensional factorial analysis (principal component analysis). The analysis of the interdimensional correlations assessed the redundancy between dimensions with expected positive correlations but which were not too high (.60-.80) [32-39]. The multidimensional analysis allowed the assessment of the gathered items with regard to different dimensions. The external validity was assessed by studying correlations between the WART and other psychological measures, such as perceived stress, well-being, or other putative addiction.

## Results

### Participants

Among the 1580 workers using WittyFit, 11.83% (187/1580) agreed to answer the WART questionnaire. Among them, 86.1% (161/187) completed the sociodemographic characteristics and the VAS. The test-retest survey was completed by 68.4% (128/187) workers. Workers’ characteristics did not differ between those who only completed the questionnaire once (*test*) and those who completed the survey twice (*test and retest*), except in tobacco consumption, with more smoker participants responding only in the test than the participants who responded both at the test and retest (32 vs 16% of smokers, *P*=.01) (Table 1).

### Results From the Work Addiction Risk Test Questionnaire

Of the 187 individuals who completed the WART questionnaire, 45.5% (85/187) were at low risk of work addiction, 32.6% (61/187) at medium risk, and 20.8 (41/197) at high risk. Women had a higher risk of work addiction than men did (27% vs 15% of workers at high risk of work addiction, *P*=.02). According to the WART, individuals exhibiting a high risk of work addiction worked for an average of 7 more hours per week than those at a low risk—46.9 (13.6) hours versus 39.4 (10.9) hours, *P*=.005.

**Table 1:** Difference between people at the test and retest in terms of sociodemographic characteristics.

Variable		Source			*P* value^a^
		Test (n=187)	Test only (n=59)	Test and retest (n=189)	
Sex, Women, n (%)		95 (50.8)	30 (52.6)	58 (55.8)	.70
Age (years), mean(SD)		41.6 (11.7)	42.0 (12.2)	41.8 (11.7)	.92
**Family situation, n (%)**					
	Single	36 (20.5)	17 (29.8)	17 (16.4)	.15
	De facto	48 (27.3)	12 (21.1)	32 (30.8)	
	Married	91 (51.7)	28 (49.1)	54 (51.9)	
	Widow(ed)	1 (0.6)	0 (0.0)	1 (1.0)	
**Education level, n (%)**					
	General Certificate of Secondary Education	2 (1.1)	1 (1.8)	1 (1.0)	.88
	General Certificate of Education–Advanced Level	8 (4.6)	4 (7.0)	4 (3.9)	
	Higher national diploma	14 (8.0)	4 (7.0)	9 (8.7)	
	Bachelor’s degree	22 (12.5)	7 (12.3)	13 (12.5)	
	Master’s degree	130 (73.9)	41 (71.9)	77 (74.0)	
**Occupational group, n (%)**					
	Merchants-business	6 (3.4)	1 (1.8)	5 (4.8)	.62
	Employees	31 (17.6)	13 (22.8)	15 (14.4)	
	Intermediate profession	12 (6.8)	3 (5.3)	7 (6.7)	
	Inactive employment	10 (5.7)	4 (7.0)	6 (5.8)	
	Manager-intellectual profession	117 (66.5)	36 (63.2)	71 (68.3)	
Hours worked per week, mean (SD)		41.6 (12.1)	42.5 (12.3)	40.8 (12.4)	.41
Seniority in the company (years), mean (SD)		10.8 (10.5)	10.9 (10.2)	11.1 (11.2)	.83
body mass index, kg.m^−2^, mean (SD)		24.2 (4.4)	25.1 (5.0)	23.7 (4.0)	.06
metabolic equivalent of task, mean (SD)		50.9 (55.5)	53.3 (53.1)	47.6 (51.4)	.44
Tobacco smoker, n (%)		39 (20.9)	19 (32.2)	20 (15.6)	.01
Alcohol users,n (%)		30 (16.0)	9 (15.3)	21 (16.4)	.84
Cannabis consumer,n (%)		14 (7.5)	5 (8.5)	9 (7.0)	.77
**WART^b^**					
	Score, mean (SD)	57.8 (11.2)	56.2 (11.6)	58.6 (11.0)	.14
	% of participants with a score <56	45.5	50.8	43.0	.51

^a^*P* value between *test only* and *test and retest*.

^b^WART: Work Addiction Risk Test.

### Acceptability

**Figure 1:**
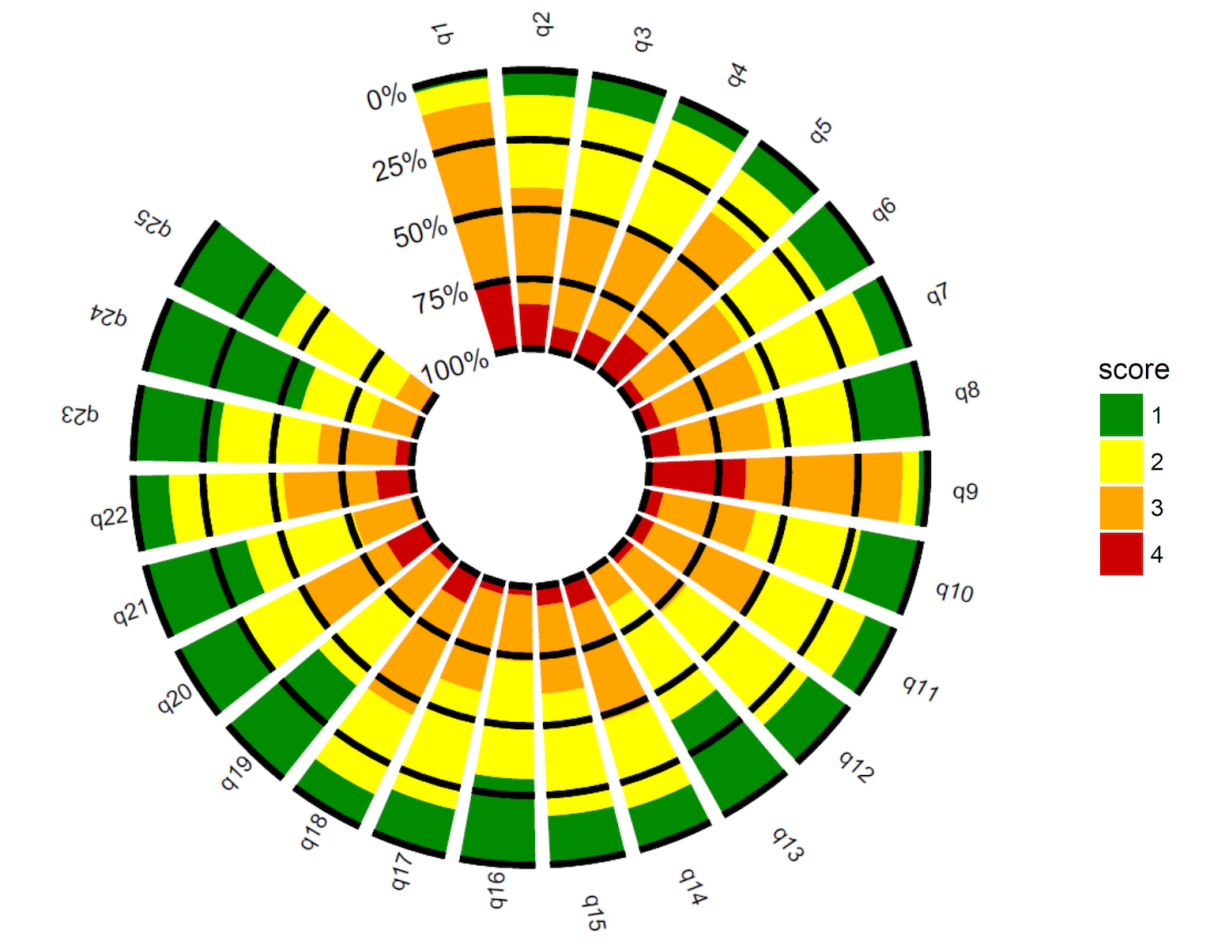
Data quality and acceptability of the French version of the Work Addiction Risk Test (WART) (n=187).

The results for data quality and acceptability of the French version of the WART are displayed in Figure 1 (see Multimedia Appendix 2). Data quality was commonly considered satisfactory if 95% of the scale was fully completed [41,42] (at least 24 of the 25 items). All of the 187 individuals who completed the WART questionnaire did so fully. In fact, no one partially replied to the questionnaire. Therefore, there were no missing data.

### Internal Validity

#### Internal Consistency

The entire WART had a Cronbach alpha of .90. The Cronbach alpha values for the various dimensions of the WART were .85 for compulsive tendencies, .82 for control, and .57 for impaired communication and self-absorption.

#### Correlation

Item-total correlation coefficients for the scale as a whole ranged from .02 to .59. Interitem correlations ranged from .23 (Questions 3 and 20) to .54 (Questions 7 and 8) for compulsive tendencies; .19 (Questions 16 and 22) to .50 (Questions 2 and 17) for control; and .08 (Questions 13 and 24) to .37 (Questions 21 and 23) for impaired communication and self-absorption. The assessment of the correlations between the questionnaire in its entirety and each dimension was statistically significant (*P*<.05) and showed that the correlation coefficient between WART and compulsive tendencies was .89; control was .84; impaired communication and self-absorption was .74; inability to delegate was .52; and self-worth was .31.

#### Principal Component Analysis

**Figure 2:**
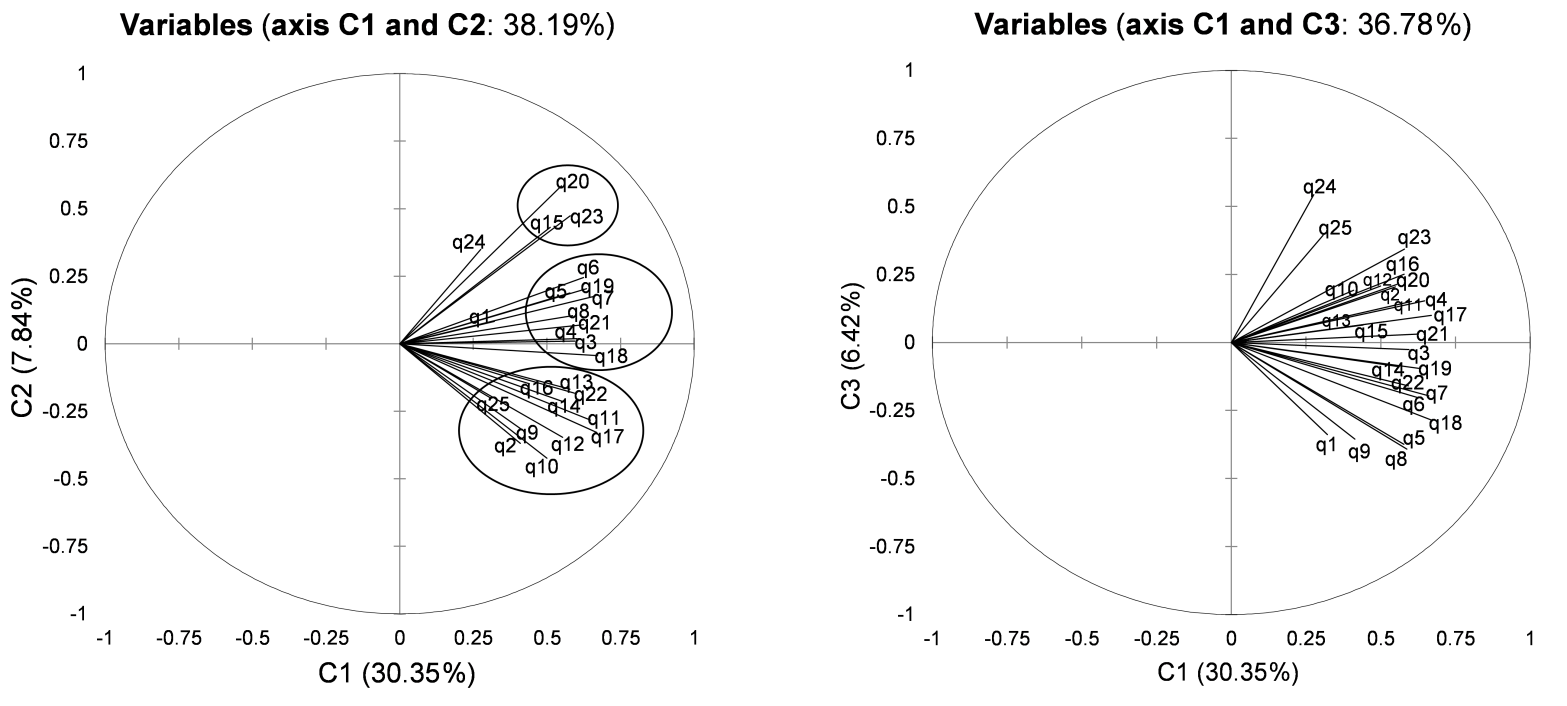
Principal component analysis: circle of correlation or the three dimensions of the French Work Addiction Risk Test (WART).

By applying the Kaiser’s criteria, in other words, the associated eigenvalues above 1 associated with a plot of the eigenvalues, we have determined four main components. Components 1 and 2 together explained the maximal variance. As presented in Figure 2, the first dimension of the French WART was associated with Component 1 and was composed of items 2, 9, 10, 11, 12, 13, 14, 16, 17, 22, and 25; the second dimension was associated with Component 2 and was composed of items 3, 4, 5, 6, 7, 8, 18, 19, and 21; and the third dimension was associated with Component 1 and was composed of items 15, 20, and 23.

#### Reproducibility

**Figure 3:**
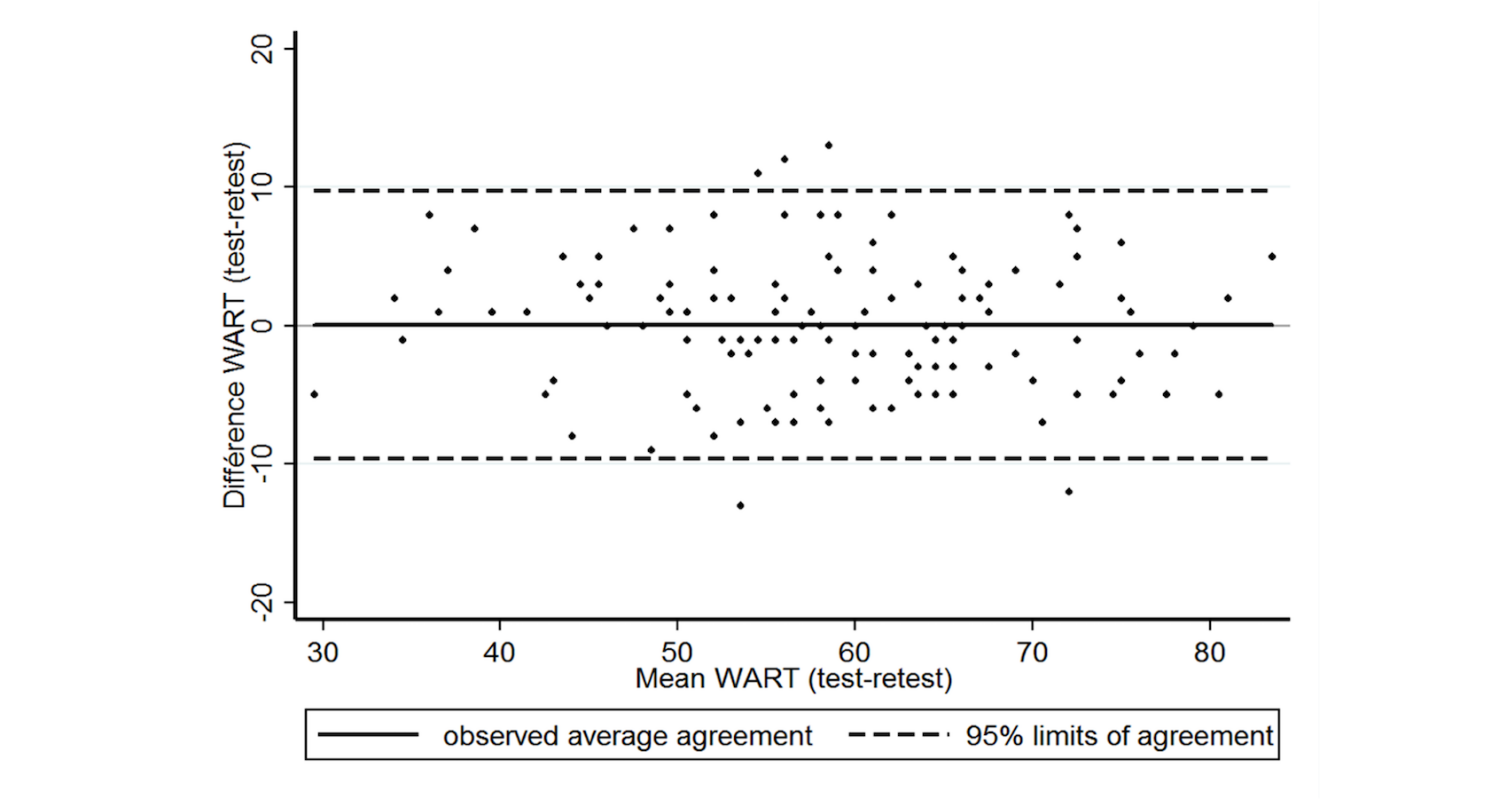
Bland and Altman plot or representation of agreement between both series of measures for the French Work Addiction Risk Test (WART).

**Figure 4:**
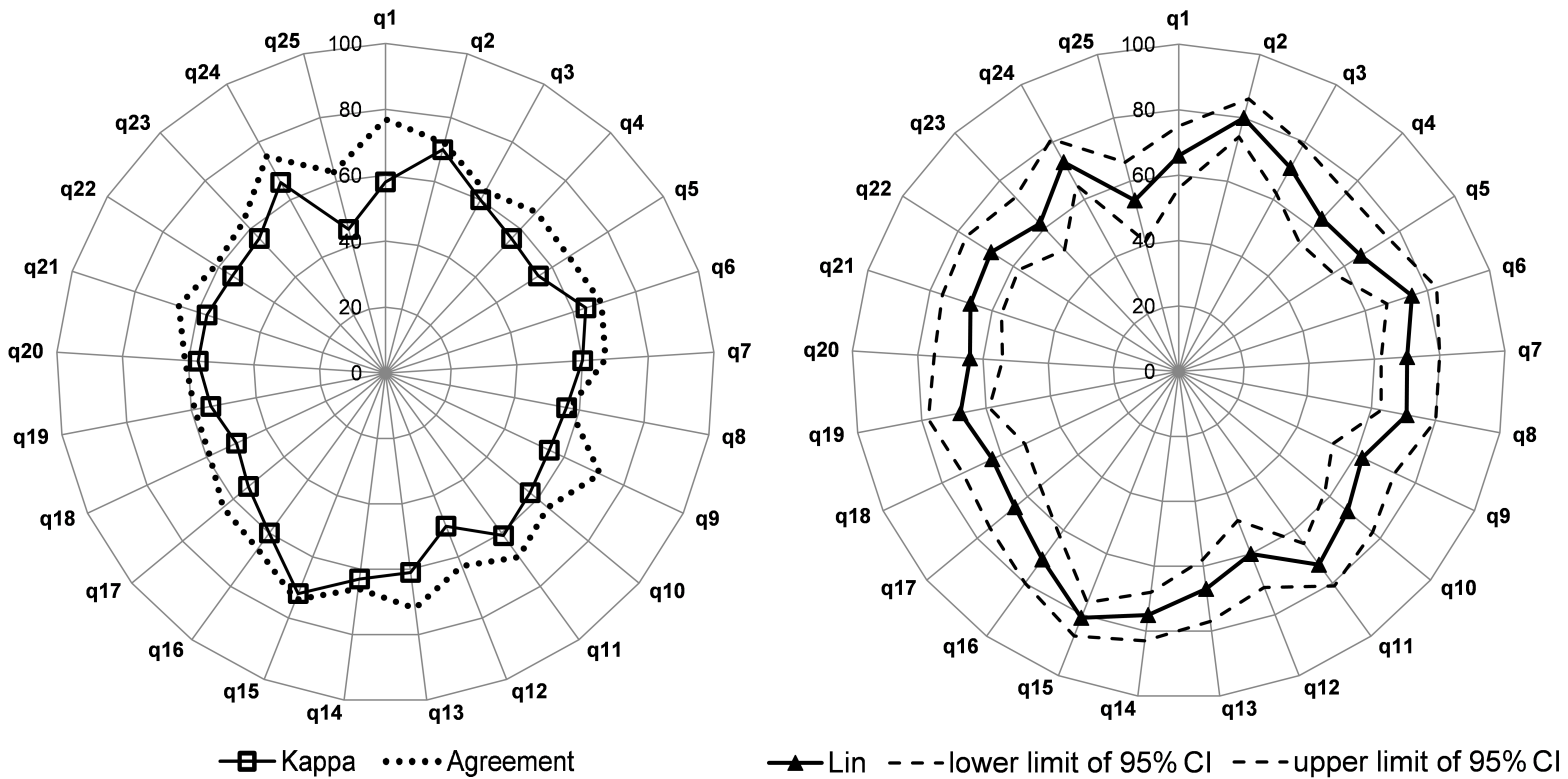
Lin concordance coefficient and Cohen kappa for each item of the Work Addiction Risk Test (WART).

The Lin concordance coefficient was .90 (95% CI 0.87-0.94) for the entire WART with a difference between the test and the retest of 0.04 (SD 4.92) (95% CI −9.61 to 9.69). The Bland and Altman plot is shown in Figure 3. For each dimension, Lin concordance coefficients were as follows: .86 (95% CI 0.82-0.91) for compulsive tendencies, .86 (95% CI 0.82-0.91) for control, .76 (95% CI 0.68-0.83) for impaired communication and self-absorption, .73 (95% CI 0.65-0.81) for self-worth, and .66 (95% CI 0.56-0.75) for the inability to delegate (Table 2). Exhaustive results for the Lin concordance coefficient and Cohen kappa for each item are shown in Figure 4 (see Multimedia Appendix 3).

### External Validity

External validity was evaluated by calculating a correlation coefficient between the WART and the others questionnaires (Table 3). The WART was well correlated to the VAS Stress at Work (coefficient correlation .43) and Stress at Home (.41) and inversely to VAS Well-being (−.40) (*P*<.05). The VAS Well-being had a reverse correlation with all dimensions of the WART (*P*<.05). The WART was poorly correlated with tobacco, alcohol, or cannabis consumption.

**Table 2:** Test-retest reproducibility for each dimension with measurement of the Lin concordance coefficient.

Dimensions	Lin concordance coefficient (95% CI)	Difference (SD), 95% CI
Compulsive tendencies	.86 (0.82-0.91)	0.09 (2.71), −5.24 to 5.41
Control	.86 (0.82-0.91)	0.02 (2.05), −4.00 to 4.03
Impaired communication and self-absorption	.76 (0.68-0.83)	0.05 (1.69), −3.27 to −3.36
Self-worth	.73 (0.65-0.81)	−0.12 (0.91), −1.91 to 1.68
Inability to delegate	.66 (0.56-0.75)	0.11 (0.49), −0.85 to 1.06

**Table 3:** External validity of the French Work Addiction Risk Test (WART), measure of correlation between WART and its dimension, and others questionnaires.

Variable	Dimensions of WART					
	WART total	Compulsive tendencies	Control	Impaired communication and self-absorption	Self-worth	Inability to delegate
Tobacco, number of cigarettes smoked per day from 0 to 30	−.10	−.10	.02	−.11	−.13	−.06
Alcohol, number of glass of alcohol per day from 0 to 8	−.12	.07	.11	.16^a^	.08	.02
Cannabis, consumption per month from 0 to 30	−.07	−.10	.02	−.05	.02	.07
Stress at work, VAS^b^ from 0 to 100	.43^a^	.41^a^	.34^a^	.32^a^	.10	.00
Stress at home, VAS from 0 to 100	.41^a^	.36^a^	.35^a^	.32^a^	.07	.08
Well-being, VAS from 0 to 100	−.40^a^	−.33^a^	−.38^a^	−.26^a^	−.17^a^	−.11^a^

^a^*P*<.05.

^b^VAS: visual analog scale.

## Discussion

This study allowed the validation of the WART questionnaire in French and focused on its acceptability, internal validity and reproducibility, construct validity, and external validity.

### Prevalence of Work Addiction and Relationships With Workers’ Characteristics

We found that 22% of the workers were at a high risk of suffering from work addiction, with predominance in women. Previous literature using WART demonstrated similar prevalence in similar populations, such as 13% of hospital doctors [12] and 22% for academic employees [14]. However, the prevalence of work addiction can be lower in other populations, such as in Italian teenagers (8%) [13], or with the use of other questionnaires (8%) [5,43]. Although some studies did not find gender differences in the prevalence of work addiction using the WART [12-14] or other questionnaires [5,43], the most recent studies report that women are at a higher risk of workaholism [23,44], which is in line with our results (27% vs 15%). This may suggest an evolution of women’s emancipation in our society, with more involvement at work [45,46]. We demonstrated that individuals with work addiction worked 7 hours more per week than those at low risk (46.9 vs 39.4 hours). The engagement in work, in terms of hours spent, is a characteristic of workaholism [6,16], with individuals devoting a majority of their time to work and working beyond what is required [6,16]. Our sensitivity analysis found no differences between the workers’ characteristics at the test and the retest, except for smoking. Among the workers responding only at the test, there were more smokers as compared with the workers responding to both the test and the retest (32% vs 16%).

The smokers could have been less motivated to respond twice, with literature suggesting a link between smoking and low levels of conscientiousness [47], impulsivity [48], lack of attention [49], impaired working memory [50], or less access to Internet [51].

### Acceptability of the French Version of the Work Addiction Risk Test

A floor or ceiling effect occurs when more than 15% of participants have the lowest or highest possible score [32,52]. A floor or ceiling effect may signify that extreme items are missing in the lower or upper end of the scale and, thus, may limit content validity. Therefore, participants with the extreme scores cannot be distinguished from each other, and reliability, as well as responsiveness is reduced because change cannot be measured in these participants [32,52]. In the French validation of the WART questionnaire, the majority of the items presented a threshold lower than 15%, as recommended in the literature [32,52]. We observed a ceiling effect for only 5 items: 1 (25% of respondents), 2 (16%), 5 (16%), 9 (35%), and 20 (16%). For example, with a possible score ranging from 1 to 4 on a 4-point Likert scale, the mean score for item 1 was 3.1 (SD 0.6) with a median of 3; 2% of responders had the lowest possible value (1) and 25% had the highest value (4). The Unfortunately, there are no studies examining the acceptability of the WART that can be used to compare our results with. So, we cannot conclude whether these results are a characteristic of our responders, a consequence of our translation, or a characteristic already present in the original English version. However, the acceptability of the French version of the WART is correct with few floor or ceiling effects. Moreover, some other well-recognized and validated questionnaires did not report acceptability in their original studies [53-57], or others reported poorer acceptability [42,58-60].

### Internal Consistency and Reproducibility

The WART’s *internal consistency* appeared satisfactory with a Cronbach alpha value, which is higher than its value in the validation of the English version (.85 [21], 0.88 [18]). The English version of the WART consists of five dimensions. We highlighted the items for compulsive tendencies (Cronbach alpha=.85), and the items for control (Cronbach alpha=.82) were closely interlinked, in line with the literature [20].

We found a high level of correlation between the overall WART score and the total score for 3 dimensions—(1) compulsive tendencies (coefficient correlation .89), (2) control (.84), and (3) impaired communication and self-absorption (.74). These three dimensions would have the greatest impact in differentiating individuals with work addiction among the population, which is in line with the literature [20]. The *reproducibility* study appeared satisfactory for the whole questionnaire, its dimensions, and the stand-alone items. Actually, the Lin concordance coefficient was .90 for the entire WART with a difference between the test and retest of 0.04 (SD 4.92), reflecting a very good reliability over time. This result was in line with the literature on the English version, which reported a Lin concordance coefficient for the test-retest of .83 at a 2-week interval [20-22,26]. Moreover, despite the fact that the literature on the English version of the WART did not report the Lin concordance coefficient for each dimension, we retrieved a Lin concordance coefficient higher than .80 for two dimensions (compulsive tendencies and control) in our French version.

### Construct Validity

Despite the fact that the French version of the WART has strong psychometric properties, results of the factorial analysis did not confirm the five dimensions of the latest study [20] but instead confirmed three dimensions. Moreover, correlations between items of each dimension remained weak, as well as a poor Cronbach alpha for items of impaired communication and self-absorption. This could be explained when examining this latest construct of the five dimensions, which was based on a low level of the correlation coefficient (.30) [20] when we used a cutoff set at .6 to .8 as described in the statistical section. Moreover, the original English version of the WART had five other different dimensions that were not drawn from a statistical approach but from the five symptoms used by clinicians for the diagnosis of workaholism: “overdoing” (Items 3, 5, 6, 7, and 15), “self-worth” (Items 8, 9, 10, 19, and 20), “control-perfectionism” (Items 1, 2, 4, 11, 12, 16, 17, 18, 21, 22, and 25), “intimacy” (Items 23 and 24), and “mental preoccupation-future reference” (Items 13 and 14) [18,21]. Those five dimensions were also not demonstrated in our analysis. However, construct differences between two language versions of the same questionnaire are common, as seen for Karasek [61,62], Hospital Anxiety and Depression Scale [60], or other questionnaires [59,63,64]. Although unlikely, translation may have changed the weight of some items. As the psychometric properties of the WART were mainly assessed on university students, our sample of French workers may emphasize some cultural specificities and work habits [65]. Cultural specificities would be investigated in subsequent work.

### External Validity

We demonstrated the relationships between the WART and VAS for “stress at work” or “stress at home” in accordance with the literature [14,15,23], and we confirmed the external validity of the French version of the WART questionnaire. The greater the risk of work addiction, the higher the stress [18]. Conversely, the well-being level was negatively correlated with the WART scores, as previously reported [2]. We did not find any significant relationships between the French-WART and tobacco, alcohol, or cannabis addictions, but to our knowledge, no studies have previously demonstrated such links.

### Limitations

The response rate may seem low compared with other studies also using a questionnaire in the French population [66-71]. However, we included a substantial sample size of workers, allowing to carry out the statistical analyses with the number of subjects required, determined a priori. Moreover, a number of respondents followed recommendations for the validation of the questionnaires [32-39]. Our sample size retrieved a sufficient prevalence of workers with a high risk of work addiction to allow a robust analysis. Despite the literature reports that a high dropout rate is inherent to this type of study with several questionnaires on the Internet [72], the number of participants who responded to both the test and retest was higher than commonly reported in the literature [60,61]. Our study may have included too many questionnaires in addition to the French version of the WART, which could have negatively affected participation [72]. Despite the observed difference of the construct, our study emphasized the excellent psychometric properties of the French version of the WART in terms of internal consistency, reproducibility, and external validity. Furthermore, there are other validated questionnaires with an internal construct differing from their original version [60-64]. We used some nonvalidated VAS. We did not control the size and type of screens used by the workers to complete the questionnaires, which may have affected our results, especially for VAS. To our knowledge, no studies have previously evaluated the influence of perception side on scores at VAS; and a study comparing answers to VAS of stress and well-being throughout different supports (paper, large computer screen, tablet, and a smartphone) is needed.

### Conclusions

The French version of the WART is a valid and reliable instrument to assess work addiction, with satisfactory psychometric properties. Used in occupational medicine, this tool would allow the diagnosis of work addiction and can be easily implemented in current practice.

## Appendix

Multimedia Appendix 1 The French version of the WART.

Multimedia Appendix 2 Data quality and acceptability of the French version of the WART.

Multimedia Appendix 3 Data of Lin concordance coefficient and Cohen kappa for each item of the WART.

## Abbreviations

ANOVA: analysis of variance
VAS: visual analog scale
WART: Work Addiction Risk Test
