# Supplementary material for: Work Addiction Test Questionnaire to Assess Workaholism: Validation of French Version
Source: JMIR Ment Health. 2018 Feb 13;5(1):e12. doi: 10.2196/mental.8215 (PMC5829463; doi:10.2196/mental.8215)
Supplement: Multimedia Appendix 1 [file mental_v5i1e12_app1.pdf]

Appendix 1. The French version of the WART.

Pas du tout vrai	Peu souvent	Souvent	Toujours vrai
**1**	**2**	**3**	**4**

1. Je préfère faire la plupart des choses moi même plutôt que de demander de l'aide	**1**	**2**	**3**	**4**
2. Je deviens très impatient lorsque je dois attendre quelqu'un ou lorsque que quelque chose prend trop de temps, comme une longue file d'attente qui avance lentement	**1**	**2**	**3**	**4**
3. Je donne l'impression d'être pressé et de toujours courir après le temps	**1**	**2**	**3**	**4**
4. Cela m'énerve d'être interrompu lorsque je suis occupé à quelque chose	**1**	**2**	**3**	**4**
5. Je me maintiens occupé et je garde toujours des choses à faire	**1**	**2**	**3**	**4**
6. Je me retrouve à faire 2 ou 3 choses en même temps, comme déjeuner et écrire un mémo tout en parlant au téléphone	**1**	**2**	**3**	**4**
7. Je m'engage excessivement en voulant faire plus que je ne peux	**1**	**2**	**3**	**4**
8. Je me sens coupable quand je ne travaille pas sur quelque chose	**1**	**2**	**3**	**4**
9. Il est important que je voie les résultats concrets de ce que je fais	**1**	**2**	**3**	**4**
10. Je suis plus intéressé par le résultat final de mon travail que par la méthode	**1**	**2**	**3**	**4**
11. Les choses ne semblent jamais avancer assez vite ou être réalisées assez rapidement pour moi.	**1**	**2**	**3**	**4**
12. Je perds mon calme quand les choses ne vont pas dans mon sens ou qu'elles ne tournent pas en ma faveur	**1**	**2**	**3**	**4**
13. Sans même le réaliser, Je repose la même question alors que je viens d'avoir la réponse une première fois	**1**	**2**	**3**	**4**
14. Je passe beaucoup de temps à tout planifier dans ma tête et à penser aux évènements futurs dans ma tête au détriment du moment présent	**1**	**2**	**3**	**4**
15. Je suis encore en train de travailler après que mes collègues aient décidés de s'arrêter	**1**	**2**	**3**	**4**
16. Je m'énerve quand les gens n'atteignent pas mes standards de perfection	**1**	**2**	**3**	**4**
17. Je m'énerve dans les situations où je n'ai pas le contrôle des choses	**1**	**2**	**3**	**4**
18. J'ai tendance à me mettre sous pression en m'imposant des délais à respecter lorsque je travaille	**1**	**2**	**3**	**4**
19. J'ai du mal à me relaxer lorsque je ne travaille pas	**1**	**2**	**3**	**4**
20. Je passe plus de temps à travailler qu'avec des amis, dans des passe-temps ou des activités de loisir	**1**	**2**	**3**	**4**
21. Je me jette dans les projets afin de prendre de l'avance avant même que les étapes aient étés définies	**1**	**2**	**3**	**4**
22. Je suis en colère contre moi même lorsque que je fais la moindre erreur	**1**	**2**	**3**	**4**
23. Je consacre plus d'idées, de temps, d'énergie à mon travail qu'à mes relations avec mon conjoint et ma famille	**1**	**2**	**3**	**4**
24. J'oublie, ignore ou minimise d'importants évènements familiaux comme les anniversaires, les fêtes ou les vacances	**1**	**2**	**3**	**4**
25. Je prends des décisions importantes avant d'avoir tous les faits et de pouvoir les examiner en détail	**1**	**2**	**3**	**4**

Calcul du total Faire la somme de chaque réponse.

Interprétation
67 à 100 = haut risque d'addiction au travail ; 57 à 66= risque moyen ; 25 à 56= risque faible.
