# Supplementary material for: Work Addiction Test Questionnaire to Assess Workaholism: Validation of French Version
Source: JMIR Ment Health. 2018 Feb 13;5(1):e12. doi: 10.2196/mental.8215 (PMC5829463; doi:10.2196/mental.8215)
Supplement: Multimedia Appendix 2 [file mental_v5i1e12_app2.pdf]

Appendix 2. Data quality and acceptability of the French version of the WART (n=187).
%, percent; Min, minimal values; Max, maximal value; n, number; SD, standard deviation.

Items	Mean	SD	Median	Min	Max	Floor Effect %	Ceiling Effect %
**1**	3.12	0.64	3	1	4	2.14	24.6
**2**	2.65	0.86	3	1	4	9.1	16.0
**3**	2.48	0.81	3	1	4	11.8	8.6
**4**	2.52	0.79	2	1	4	8.1	10.7
**5**	2.74	0.87	3	1	4	11.8	16.0
**6**	2.31	0.84	2	1	4	19.3	4.8
**7**	2.40	0.77	2	1	4	11.2	6.4
**8**	2.30	0.97	2	1	4	25.1	11.2
**9**	3.22	0.70	3	1	4	3.2	34.8
**10**	2.24	0.86	2	1	4	21.9	5.9
**11**	2.44	0.77	2	1	4	11.2	5.9
**12**	2.13	0.74	2	1	4	18.2	3.7
**13**	1.75	0.69	2	1	3	39.0	14.4
**14**	2.46	0.87	3	1	4	15.5	9.6
**15**	2.28	0.83	2	1	4	17.7	7.0
**16**	1.99	0.82	2	1	4	31.0	3.2
**17**	2.26	0.78	2	1	4	17.1	3.7
**18**	2.54	0.86	3	1	4	12.8	11.2
**19**	1.86	0.88	2	1	4	42.8	4.3
**20**	2.40	1.04	3	1	4	26.2	15.5
**21**	1.87	0.80	2	1	4	37.4	1.6
**22**	2.46	0.88	2	1	4	12.8	12.8
**23**	2.09	0.90	2	1	4	30.5	5.9
**24**	1.60	0.79	1	1	4	57.8	1.6

**25**	1.73	0.7<br>1	2	1	4	40.6	1.6
