# Supplementary material for: Work Addiction Test Questionnaire to Assess Workaholism: Validation of French Version
Source: JMIR Ment Health. 2018 Feb 13;5(1):e12. doi: 10.2196/mental.8215 (PMC5829463; doi:10.2196/mental.8215)
Supplement: Multimedia Appendix 3 [file mental_v5i1e12_app3.pdf]

Appendix 3. Lin concordance coefficient and Cohen's kappa for each item of the WART.
95% CI, 95% confidence interval; %, percent.

	Cohen's kappa (% agreement)	Lin concordance coefficient (95% CI)
**Q1**	0.56 (77.3%)	0.66 (0.56–0.75)
**Q2**	0.70 (71.9%)	0.80 (0.74–0.86)
**Q3**	0.60 (63.3%)	0.71 (0.62–0.79)
**Q4**	0.56 (67.2%)	0.65 (0.54–0.75)
**Q5**	0.55 (65.6%)	0.66 (0.56–0.76)
**Q6**	0.64 (68.8%)	0.75 (0.67–0.83)
**Q7**	0.60 (67.2%)	0.70 (0.62–0.79)
**Q8**	0.56 (57.0%)	0.71 (0.63–0.80)
**Q9**	0.55 (71.9%)	0.62 (0.52–0.73)
**Q10**	0.57 (64.1%)	0.67 (0.58–0.77)
**Q11**	0.61 (68.8%)	0.73 (0.65–0.81)
**Q12**	0.50 (63.3%)	0.60 (0.49–0.71)
**Q13**	0.61 (71.9%)	0.67 (0.58–0.77)
**Q14**	0.63 (66.4%)	0.75 (0.68–0.83)
**Q15**	0.72 (74.2%)	0.81 (0.76–0.87)
**Q16**	0.60 (66.4%)	0.71 (0.62–0.80)
**Q17**	0.54 (64.1%)	0.65 (0.55–0.75)
**Q18**	0.50 (58.6%)	0.63 (0.52–0.73)
**Q19**	0.54 (59.4%)	0.68 (0.59–0.78)
**Q20**	0.57 (60.9%)	0.65 (0.54–0.75)
**Q21**	0.57 (66.4%)	0.67 (0.57–0.76)
**Q22**	0.55 (60.9%)	0.68 (0.58–0.80)
**Q23**	0.56 (63.3%)	0.62 (0.51–0.73)
**Q24**	0.66 (75.0%)	0.73 (0.65–0.81)
**Q25**	0.45 (63.3%)	0.54 (0.41–0.70)
